# Metabolic modeling predicts unique drug targets in *Borrelia burgdorferi*

**DOI:** 10.1128/msystems.00835-23

**Published:** 2023-10-19

**Authors:** Peter J. Gwynne, Kee-Lee K. Stocks, Elysse S. Karozichian, Aarya Pandit, Linden T. Hu

**Affiliations:** 1 Graduate School of Biomedical Sciences, Tufts University School of Medicine, Boston, Massachusetts, USA; 2 Tufts Lyme Disease Initiative, Tufts University, Boston, Massachusetts, USA; University of North Carolina at Charlotte, Charlotte, North Carolina, USA

**Keywords:** antimicrobial agents, Lyme disease, drug discovery, host-pathogen interactions, metabolic modeling

## Abstract

The Lyme disease bacterium *Borrelia burgdorferi* is extremely host dependent, with a small genome and correspondingly limited metabolism. As such, it is an excellent candidate for the development of targeted, narrow-spectrum antimicrobials. To accelerate drug discovery in this fastidious bacterium, *in silico* genome-scale metabolic modeling was used to construct a map of *B. burgdorferi*’s metabolism. This map was used to predict essential genes and enzymes; experimental data validated these predicted hits as viable drug targets. Repurposing existing small-molecule inhibitors, it is shown that inhibition of two predicted essential enzymes (pyridoxal kinase and serine hydroxymethyltransferase) selectively kills *B. burgdorferi* in culture. Thus, the essential processes identified here represent targets for the development of narrow-spectrum antimicrobials. This pipeline, pairing *in silico* discovery with validation in culture, may be useful for other genetically intractable pathogens.

**IMPORTANCE:**

Lyme disease is often treated using long courses of antibiotics, which can cause side effects for patients and risks the evolution of antimicrobial resistance. Narrow-spectrum antimicrobials would reduce these risks, but their development has been slow because the Lyme disease bacterium, *Borrelia burgdorferi*, is difficult to work with in the laboratory. To accelerate the drug discovery pipeline, we developed a computational model of *B. burgdorferi*’s metabolism and used it to predict essential enzymatic reactions whose inhibition prevented growth *in silico*. These predictions were validated using small-molecule enzyme inhibitors, several of which were shown to have specific activity against *B. burgdorferi*. Although the specific compounds used are not suitable for clinical use, we aim to use them as lead compounds to develop optimized drugs targeting the pathways discovered here.

## INTRODUCTION

*Borrelia burgdorferi* has a small genome of ~1.5 Mbp (1–3) and, as a result, is highly dependent on its tick and vertebrate hosts for the provision of many metabolites (4–6). Small genomes by necessity have few redundant pathways or enzymes and a higher proportion of essential genes. The minimal genome of *B. burgdorferi*, therefore, makes it an attractive candidate for the development of narrow-spectrum antibiotics targeting these essential genes.

While the bacterium does not live independently of its hosts in the wild, it is culturable in the laboratory. The organism is extremely fastidious, however, and requires a complex and undefined growth medium. The standard *in vitro* growth medium Barbour-Stoenner-Kelly (BSK) contains numerous undefined components: yeast extract, bovine serum albumin, and rabbit serum (7, 8). Traditional metabolic and genetic studies are further hindered by the species’ slow growth rate (9) and a complex and unstable genome (10, 11). To circumvent the challenges of *in vitro* metabolic studies, we have developed a genome-scale metabolic model for *B. burgdorferi* B31. Genome-scale metabolic modeling permits the creation and analysis of *in silico* simulations of metabolism, as predicted by comparison of an organism’s protein coding sequences with those of known metabolic enzymes (12). Similar models are often used for the optimization of pathways and strains in the bioproduction of high-value metabolites (13–15) but have also been applied to the study of pathogenesis and host-pathogen interactions, particularly in intractable organisms (16, 17).

In addition to describing the basic metabolic capacity of the organism, such models can be used to predict essential processes. The prediction of essential reactions by genome-scale metabolic models has allowed for the identification of drug targets and the design of novel drugs in both prokaryotic (18) and eukaryotic (19) systems. Comparison of the new *B. burgdorferi* metabolic model with existing models for well-characterized pathogens (*Escherichia coli* and *Staphylococcus aureus*) permits the selection of targeted antimicrobials with specific activity against *B. burgdorferi*.

Current clinical guidelines recommend treatment of Lyme borreliosis with antibiotics (penicillins, tetracyclines, or macrolides) with risks of nosocomial infections such as *Clostridiodes difficile* due to disruption of the native microbiome (20, 21). Although antimicrobial resistance is rarely described in *Borrelia*, the use of broad-spectrum antibiotics [and, in particular, extended treatment regimens (22)] may also drive enduring resistance in off-target bacteria (23). Lyme disease prophylaxis is currently limited to the use of antibiotics within a narrow window after a high-risk tick bite (24). An alternative means of reducing infections is the reduction of carriage in wild animal reservoirs. Previous studies treating small mammals with doxycycline were highly effective in reducing *Borrelia* and *Anaplasma* infections (25, 26), but concerns around antimicrobial resistance have prevented widespread implementation.

The identification and use of narrow-spectrum antibiotics [such as the recently rediscovered hygromycin A (27)] with specific activity against the Lyme disease *Borrelia* would minimize the potential harms of prolonged broad-spectrum antibiotic use. In addition to reducing the risk of antimicrobial resistance in off-target commensals, targeted anti-borrelials could also be used prophylactically by high-risk individuals or to reduce infections by eradication of the disease from its wild animal reservoirs. We describe the construction, analysis, and validation of a metabolic model for *B. burgdorferi* for the prediction of critical reactions which are targets for the development of narrow-spectrum anti-borrelials.

## MATERIALS AND METHODS

### Metabolic model construction

The complete *B. burgdorferi* B31 genome [GenBank assembly GCF_000008695.2 (2, 3)] of a ~900-kbp chromosome and 22 plasmids was used throughout. The metabolic model iBB151 was constructed using RAVEN 2.5.3 (28), chosen for its ability to integrate models derived from multiple sources. Reconstruction and analysis were performed in MATLAB R2020a. In absence of a scaffold model from a close genetic relative, three models were created by different methods and integrated. Two models were derived from existing metabolic databases KEGG (RAVEN command *getKEGGModelForOrganism*) and MetaCyc (RAVEN command *getMetaCycModelForOrganism*). The KEGG database identifies metabolic functions from coding sequences based on similarity to characterized enzymes (29). KEGG contains automated annotations of over 5,000 genomes, including that of *B. burgdorferi* B31, with associated reactions and metabolites. MetaCyc is a curated database of metabolic enzymes, reactions, and metabolites (30). These curated enzyme sequences were queried using the *B. burgdorferi* B31 genome to generate *de novo* a draft model structure.

The RAVEN 2.5 package also includes a series of hidden Markov models (HMMs) trained using HMMER3 (31) on nonredundant protein sequences derived from the KEGG database (version 91.0) and arranged in nonredundant clusters by CD-HIT (32). Three HMMs were used to generate draft models based on similarity to KEGG orthologues (RAVEN command *getKEGGModelForOrganism*) with redundancy cutoffs of 50%, 90%, and 100%. These three models were merged first with each other and then the KEGG model (RAVEN command *mergeModels*) and, finally, with the MetaCyc model (RAVEN command *combineMetaCycKEGGModels*) to produce the combined draft model containing 378 metabolic reactions. Manual curation removed duplicates, unconnected reactions, and obvious mis-annotations and added processes described experimentally but not predicted by any of the pipelines (33). With few transporters described or predicted in *B. burgdorferi*, transport and exchange reactions were added to allow every reaction in the model to carry flux (as determined by the RAVEN command *hasFlux*). Memote (34) was used to confirm stoichiometric consistency. The final Memote score was 87%.

### Biomass reaction

*In silico* “growth” is represented by flux through a pseudo-reaction which consumes known biomass precursors. Biomass composition has not been extensively studied in *B. burgdorferi*. In the absence of a well-studied close relative of *B. burgdorferi*, the biomass objective function was derived from that of that of *E. coli* [as described in the model iAF1260 (35)], with modifications based on available literature. Total lipid composition and contribution to dry weight were adjusted based on experimental data (36), and lipopolysaccharide was removed from the biomass calculations. The contribution of the remaining major biomass constituents (DNA, RNA, protein, and peptidoglycan) were adjusted to account for the higher amount of lipid in *B. burgdorferi* dry weight. Codon utilization (amino acid composition) and GC content were determined from the genome using CoCoPUTs (37). Metal ions were omitted, as were any cofactors not predicted elsewhere in the model including thiamin, which is not detectable in *B. burgdorferi* (38).

### Analysis of iBB151

Analysis was performed using the COBRA toolbox v3.0 (39) to determine flux through each reaction while maximizing for a single objective function. For all analyses, the objective function was the reaction EX_BM, the export of biomass. Essential reactions were identified using the Cobra function *singleRxnDeletion*, which sequentially inactivates each reaction and attempts to solve the model for the objective function. Any inactivation which produces an insoluble model (i.e., a growth rate of 0) is classed as essential. Synthetic lethal single and double gene deletions were similarly predicted using *singleGeneDeletion* and *doubleGeneDeletion*. For comparison with other organisms, the same method was used on existing *E. coli* and *S. aureus* metabolic models iML1515 (40) and iYS854 (41).

### Minimum inhibitory concentrations

Inhibitors of predicted essential reactions were identified by literature search. These were tested against *B. burgdorferi* by growth BSK II medium, composed of bovine serum albumin (50.00 g/L), CMRL-1066 [US Biologicals (9.80 g/L)], HEPES (6.60 g/L), peptone (5.60 g/L), dextrose (5.60 g/L), sodium bicarbonate (2.44 g/L), yeast extract (2.20 g/L), sodium pyruvate (1.00 g/L), sodium citrate (0.90 g/L), N-acetyl glucosamine (0.50 g/L), and 6.2% rabbit serum. Media were filter sterilized, and the pH was adjusted to 7.6 before the addition of gelatin to 1.4% and sterile water to 1 L and stored at −20°C. The *B. burgdorferi* B31 isolate used throughout this study was an infectious strain, cultured from the ear of an infected C3H-HeJ mouse. By PCR plasmid typing, this strain possessed all B31 plasmids except lp5, cp32-6, and cp32-9, which carry no metabolic genes either described previously or predicted in the modeling above.

Minimum inhibitory concentration (MIC) was determined for three drugs: cycloserine (Thermo Scientific) at 0–256 μg/mL, theophylline (TCI Chemicals), and pemetrexed hydrate (TCI Chemicals) at 0–8 mg/mL. Cycloserine was diluted 1:500 from a stock (in water) of 128 mg/mL: an equivalent volume of water was added to no-drug controls. Theophylline and pemetrexed were dissolved directly into BSK or tryptic soy broth (TSB) medium, which was then filter sterilized with a 0.22-µm nitrocellulose syringe filter. Dilution series were inoculated with 1 × 10^5^ cells/mL *B. burgdorferi* B31 from an early stationary phase (1 × 10^8^ cells/mL) culture. Cultures were grown in volumes of 200 µL in uncoated flat-bottom 96-well plates at 32°C with 1% CO_2_ for 5–7 days, until drug-free controls reached at least 1 × 10^8^ cells/mL. Cells were directly enumerated using a Petroff-Hausser counter (Hausser Scientific). *E. coli* MG1655 and *S. aureus* 502a were cultured in TSB (Becton Dickinson): tryptone (17.0 g/L), soytone (3.0 g/L), glucose (2.5 g/L), sodium chloride (5.0 g/L), and dipotassium phosphate (2.5 g/L). Overnight cultures were diluted 1:1,000 into fresh media containing the same dilutions series of drug and grown overnight (18 hours) at 37°C. Growth was measured by optical density at 600 nm (OD_600_) using a plate reader (Biotek Synergy H1). The MIC was defined as the lowest concentration at which growth was reduced by 90% or more compared to an untreated control. Growth data presented are the average of biological triplicates in all cases.

### Measurement of bacterial viability

Triplicate *B. burgdorferi* B31 cultures were grown to exponential phase (6 × 10^6^–2 × 10^7^) or stationary phase (>9 × 10^7^). Five-hundred-microliter culture was pelleted (10,000 g, 5 minutes) and resuspended in 500-µL fresh BSK containing theophylline (8.0 and 1.0 mg/mL), pemetrexed (4.0 and 0.5 mg/mL), or doxycycline (1.0 µg/mL). Untreated controls were resuspended in BSK only. All conditions were incubated for 20 hours at 32°C with 1% CO_2_. For enumeration of colony-forming units, cultures were plated in overlay agars onto BSK composed as above but for the omission of gelatin and the addition of agarose to 0.65% to solidify the media. Overlays that were 1.8 mL were plated onto 5-mL plates in 60-mm Petri dishes. Treated cultures were diluted 10^−5^ or 10^−6^ into a final volume of 900 µL, to which 900-µL 1.7% low-melting point agarose (Apex) was added. The lower limit of detection was, therefore, 2 × 10^5^ cells or a 99.8% reduction from a 1 × 10^8^ starting culture.

For live/dead staining, 100-µL cell culture was mixed with 100-µL BacLight LIVE/DEAD stain (Invitrogen L7007) and incubated in the dark at room temperature for 15 minutes. In addition to the untreated control, a heat-killed control culture was pelleted as above and resuspended in 500-µL saline (0.9% NaCl, pH 7.4). Two-hundred-microliter cell suspension was incubated at 70°C for 10 minutes. Five-microliter stained cells were visualized on slides using an Echo Revolve fluorescence microscope fitted with darkfield, FITC (Ex: 470/40, Em: 525/50) and TXRED (Ex: 560/40, Em: 630/75) filter cubes. In addition to the untreated control, a heat-killed control culture was pelleted as above and resuspended in 500-µL saline (0.9% NaCl, pH 7.4). Two-hundred-microliter cell suspension was incubated at 70°C for 10 minutes. The FITC channel detected SYTO 9-stained (viable) cells, while the TXRED channel detected propidium iodide-stained (dead) cells. FITC and TXRED images were contrast enhanced and merged (in ImageJ), and fields were counted until a minimum of 200 cells were counted in a single channel. The same images were then counted for the second color, with percentage killing calculated as TXRED cells/(TXRED cells + FITC cells) × 100.

## RESULTS

### iBB151, the metabolic model of *Borrelia burgdorferi*

**Fig 1:**
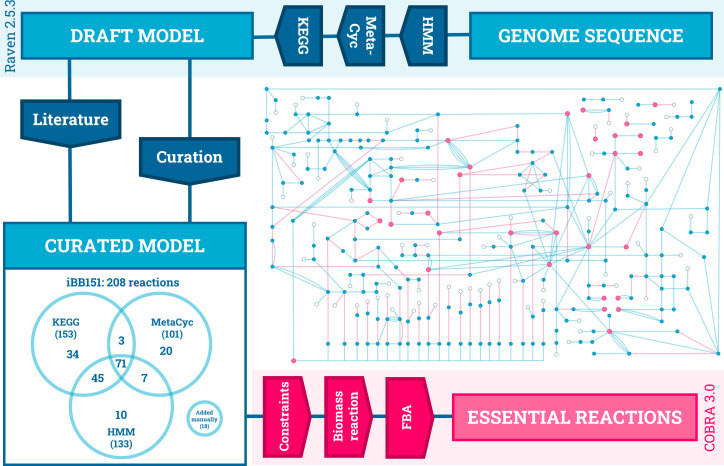
Generation and analysis of the genome-scale metabolic model of *B. burgdorferi* iBB151. Predicted coding sequences from the genome were assigned enzymatic functions by comparison to two databases (KEGG and MetaCyc) and by a *de novo* annotation using pre-trained HMMs. The curated model mapped a total of 208 enzymatic reactions to 151 genes and was used to predict reaction essentiality by flux balance analysis (FBA). Eighty-five transport reactions provide extracellular metabolites (hollow circles), to 213 cytoplasmic reactions involving 232 cytoplasmic metabolites (blue circles). Thirty-two biomass metabolites (pink circles) produced by 77 essential reactions (pink lines) are consumed in the biomass reaction. A larger, labeled version of the inset metabolic map is available in supplemental file S2.

iBB151, an *in silico* model of *B. burgdorferi* metabolism, was generated by combining three genome annotation pipelines with experimental data from the literature (Fig. 1). *B. burgdorferi* B31 has a small genome, with a chromosome of <1 Mbp and plasmids totaling a further 600 kbp, and the model created is correspondingly small, describing only 208 metabolic reactions. A total of 151 genes were annotated here, representing 10.5% of the predicted protein coding sequences of the genome. Reaction and gene annotations are cross-referenced in the “Rxns” tab of the model file, supplemental file 4. This small model is reflective of a genome with a high proportion of hypothetical genes with no similarity outside of the genus and a significant proportion of protein-coding genes devoted to (nonmetabolic) surface lipoproteins (42–44). A total of 388 reactions (metabolic, transport, exchange, and biomass reactions) are modeled in iBB151. The previous *E. coli* and *S. aureus* models used as controls here model 2,713 and 743 reactions, respectively. The size and complexity of metabolic models reflect the size of the target genome but are also dependent on the degree of conservation with well-characterized reference genomes, as annotations are based on homology to known enzymes. Given the evolutionary divergence of the spirochetes and limited experimental data (33), the value of combining multiple annotations is demonstrated by Fig. 1. Only 71 reactions were predicted by all three annotation methods, while each pipeline contributed numerous unique reactions. The model is available in Microsoft excel format in the supplemental information (supplemental file S4), along with image files depicting model overviews (files S2 and S3). An SBML (version 3.1) file is available at https://github.com/pjgwynne/iBB151.

The overall architecture of the model features two compartments, extracellular and cytoplasmic. Insufficient experimental data exist for *B. burgdorferi* to confidently predict localization to the periplasmic space. Extracellular metabolites mimic the growth medium and are transported into the cell by transport reactions. Cytoplasmic reactions convert metabolites into biomass metabolites, which are consumed by a biomass reaction to model growth. Many major metabolic pathways are missing from *B. burgdorferi*. As in previous predictions (1, 2), no enzymes of the tricarboxylic cycle, oxidative phosphorylation, or fatty acid or sterol synthesis are found. Amino acid metabolism is largely absent, as is the urea cycle and polyamine synthesis. Energy is generated through glycolysis, with lactic acid the terminal product (file S3: ‘RXNS’: BB_0087). As demonstrated by experimental data (45, 46) glucose (BB_0730), mannose (BB_0407), glycerol (BB_0241)_, and disaccharides including chitobiose (BB_0620) can be utilized via glycolysis. Other predictions also match available experimental data: N-acetyl glucosamine and N-acetyl mannosamine can be used interchangeably (BB_0644) (47). Acetyl-CoA initiates a partial mevalonate pathway (BB_0685) essential for the synthesis of peptidoglycan precursor undecaprenyl phosphate (48). The arginine deiminase system produces ornithine (BB_0842) (49), which is the peptidoglycan crosslinking diamino acid (50, 51). Again, matching recent experimental data, two glycerol-3-phosphate dehydrogenases were predicted: one using NADH as a cofactor (BB_0368) (52) and one using FADH (BB_0243) (53).

### Prediction of essential reactions

Essential reactions were predicted by sequential inactivation of each reaction and solving with growth (reaction EX_BM) as the objective function. Growth rate of the wild type (WT) model in complete media was 0.274 mmol/gDW/h, with all single reaction deletions either having no effect or completely preventing growth. Seventy-seven single reactions were essential for growth, representing 37.0% of the 208 metabolic reactions described. A similar percentage of transport reactions were essential (32/85, 37.6%). The growth rate and growth ratio (growth rate of mutant/growth rate of wild type) after inactivation of each individual reaction are available in supplemental file S5, “SRD,” which lists the growth rate of a model in which each reaction is deleted. Essential reactions are those where the model cannot be solved, and the growth rate is 0. As expected in a small model, 37% is a relatively high percentage of essential reactions. Similar screens of metabolic reactions using models of *E. coli* (40) and *S. aureus* (41) predicted 227/1,552 (14.6%) and 435/1,455 (29.8%) essential reactions, respectively. A complete list of essential reactions is included in the supplemental information (file S5: SRD).

Transport processes are poorly understood in *B. burgdorferi*. Although a small number of transport reactions were predicted by the genome reconstruction pipeline, most were added manually to allow all predicted reactions to carry flux. Thus, 85 transport reactions were required to allow every reaction in the model to function; the complete list of these can be found in supplemental file S4, RXNS tab. Transport of 85 metabolites allowed the full function of the model, but only 32 transport reactions were found to be required for the production of biomass in the above screen of essential reactions. These essential nutrients reflect those which have been described experimentally, including most amino acids (54), lipid precursors (55), and nucleotides (56) as well as a number of cofactors and vitamins for which *B. burgdorferi* has no predicted synthetic pathways.

A transposon mutagenesis screen was previously completed in *B. burgdorferi* (57). Although the unsaturated library cannot confidently predict gene essentiality, any gene with a transposon insertion can be assumed to be nonessential for growth in complex medium. Overall, the previous library featured insertions in 45.5% of protein-coding genes (57). Of the 151 genes annotated in iBB151, only 25.8% were previously inactivated in the transposon library, likely reflecting the immutability of core metabolic genes. The transposon library is cross-referenced to the predictions of essentiality in iBB151 in supplemental file S5, SGD. Most of the transposon mutants were found in genes predicted to be nonessential: mutability of the predicted nonessential genes was 38.8%. Growth rates and growth ratios (growth rate of deletion/growth rate of wild type) of each mutant are available in supplemental file S5, SGD tab. As above, a mutant with a growth rate less than the WT (growth ratio <1) is impaired. A mutant resulting in a growth ratio of 0 is lethal.

Only six predicted essential genes (6/66, 9.1% mutability) (BB_0377, BB_0527, BB_0585, BB_0605, BB_0767, and BB_0841) had transposon insertions. Three of these insertions were at the end of the gene (insertion ratio ≥0.97) and thus may not have been inactivating insertions. BB_0377 encodes ribosylhomocysteine lyase, which is essential to avoid the accumulation of waste product S-ribosyl homocysteine *in silico. In vivo*, this product may be excreted or detoxified elsewhere in the absence of the BB_0377 gene product. BB_0841 is part of the pathway producing the cell wall diamino acid ornithine. This gene may be mutable in cultured *Borrelia*, where ornithine can be imported from the growth medium, but is essential *in silico* without this compensatory transport reaction. The product of BB_0605 is predicted to be essential for cell wall maturation but was previously shown to have five transposon insertions. This gene is evidently mis-annotated in iBB151, but no alternative prediction was found.

Pairs of lethal gene deletions may represent targets for combination therapies but are also illustrative of important pathway intersections and rare examples of metabolic redundancy. For instance, BB_0137 and BB_0593 are nonessential individually but lethal in combination. These two genes encode fatty acid ligases producing acyl-CoA and acyl-acyl carrier protein (ACP), respectively. Lethality in the double deletion highlights the importance of fatty acid scavenging. A grid of double gene deletions is shown in supplemental file S5, “DGD.” Unlike the single reaction or single gene deletions, many double gene deletions result in intermediate growth phenotypes (growth ratios >0 but <1).

### Identification of anti-borrelial targets

Many of the 77 reactions predicted as essential for growth are the targets of existing antibiotics. These include cell wall synthesis (58) (12 reactions), aminoacyl tRNA ligases (59) (19 reactions), and alanine racemase (60). Others such as the mevalonate pathway (61) (eight reactions) and glutamate racemase (62) have been proposed as targets for the development of novel antibiotics. This *in silico* screen also predicts the efficacy of compounds targeted at specific borrelial pathways. Adenosylhomocysteine nucleosidase has been extensively characterized as a unique target for the development of drugs with specific activity against *B. burgdorferi* (63, 64). Similarly, the essentiality of a mammalian-type mevalonate pathway for the synthesis of undecaprenyl phosphate is consistent with prior studies of this pathway (65).

In order to identify enzymes whose inactivation is lethal only in *B. burgdorferi*, the predicted essential reactions from iBB151 were compared with those of iML1515 (40) (*E. coli*) and iYS854 (41) (*S. aureus*). Twenty-one reactions were predicted to be essential only in *B. burgdorferi*. A further seven were predicted to be partially specific: essential in only one of the comparator species. These 28 potential narrow-spectrum drug targets are listed in Table 1.

**TABLE 1:** Selected essential enzymatic reactions in *B. burgdorferi^a^*

*B. burgdorferi* (iBB151)						*E. coli* (iML1515)		*S. aureus* (iYS853)	
Pathway	Reaction ID	Full name	Reaction	Gene	Deletion growth ratio	Reaction ID	Deletion growth ratio	Reaction ID	Deletion growth ratio
	ARG_D	Arginine deiminase	L-arginine + H2O ⇌ ammonium + L-citrulline	BB_0841	**0.00**	nf	–	ARGDr	1.00
	SAH_N	Adenosyl- homocysteine nucleosidase	S-adenosylhomocysteine + H2O → adenine + S-ribosyl-L-homocysteine	BB_0375/BB_0588/BB_I06	**0.00**	AHCYSNS	**0.00**	HCYSNS	1.00
	PYDX_K	Pyridoxal kinase	ATP + pyridoxal → ADP + H+ + pyridoxal 5'-phosphate	BB_0768	**0.00**	PDXK	1.00	PYDXK_1	1.00
Folates	METTHF_DH	Methylene-THF dehydrogenase	Methylene-THF + NADP+ ⇌ methenyl-THF + NADPH	BB_0026	**0.00**	MTHFD	0.99	MTHFD	0.98
	MENTHF_CH	Methylene-THF cyclohydrolase	Methenyl-THF + H2O ⇌ formyl-THF + H+	BB_0026	**0.00**	MTHFC	0.99	MTHFC	0.98
	SER_HMT	Serine hydroxymethyl- transferase	L-serine + THF ⇌ glycine + methylene-THF + H2O	BB_0601	**0.00**	GHMT2r	0.99	GHMT2r	0.98
Glycolysis	GAP_DH	GAP dehydrogenase	Glyceraldehyde 3-phosphate + NAD+ + phosphate ⇌ 3-phospho-glyceroyl-phosphate + NADH+ H+	BB_0057	**0.00**	GAPD	0.87	GAPD_1	0.00
	PGLYC_K	Phosphoglycerate kinase	ATP + 3-phospho-glycerate ⇌ ADP + 3-phospho-glyceroyl-phosphate	BB_0055/BB_0056	**0.00**	PGK	0.87	PGK	0.00
	PGLYC_M	Phosphoglycerate mutase	2-Phospho-glycerate ⇌ 3-phospho-glycerate	BB_0658	**0.00**	PGM	0.93	PGM	0.78
	ENO	Enolase	2-Phospho-D-glycerate ⇌ phosphoenolpyruvate + H2O	BB_0337	**0.00**	ENO	0.92	ENO	0.78
Lipids	UGLUC_E	UDP-glucose epimerase	UDP-glucose ⇌ UDP-galactose	BB_0444	**0.00**	UDPG4E	1.00	UDPG4E	1.00
	PC_S	Phosphatidyl- choline synthase	CDP-diacylglycerol + choline → CMP + phosphatidylcholine + H+	BB_0249	**0.00**	nf	–	nf	–
	DAG_GT	Diacylglycerol galactosyl- transferase	CDP-diacylglycerol + UDP-galactose ⇌ galactosyldiacylglycerol + UDP	BB_0454	**0.00**	nf	–	nf	–
	CHO_GT	Cholesterol galactosyl- transferase	Cholesterol + UDP-galactose ⇌ galactosylcholesterol	BB_0572	**0.00**	nf	–	nf	–
Mevalonate	ACTCOA_AT	Acetyl CoA acetyltransferase	Two acetyl-CoA ⇌ acetoacetyl-CoA + CoA	BB_0685	**0.00**	ACACT1r	0.99	ACACT1r	**0.00**
	HMGCOA_S	Hydroxymethyl-CoA synthase	Acetoacetyl-CoA + acetyl CoA + H2O ⇌ (S)-3-hydroxy-3-methylglutaryl-CoA + CoA + H+	BB_0683	**0.00**	nf	–	HMGCOAS	–
	MEV_K	Mevalonate kinase	ATP + mevalonate ⇌ ADP + mevalonate 5-phosphate + H+	BB_0688	**0.00**	nf	–	MEVK1_1,/MEVK2/MEVK3/MEVK4^*b*^	**0.00**
	PMEV_K	Phospho-mevalonate kinase	ATP + mevalonate 5-phosphate ⇌ ADP + mevalonate diphosphate	BB_0687	**0.00**	nf	–	PMEVK	**0.00**
	PPMEV_DC	Mevalonate diphosphate decarboxylase	ATP + mevalonate diphosphate → ADP + CO2 + isopentenyl diphosphate + phosphate	BB_0686	**0.00**	nf	–	DPMVD	**0.00**
Nucleotides	IPP_I	Isopentenyl diphosphate isomerase	Isopentenyl diphosphate ⇌ dimethylallyl diphosphate	BB_0684	**0.00**	IPDDI	1.00	IPDDI	**0.00**
	DCYT_K	Deoxycytidine kinase	2'-Deoxycytidine + ATP → dCMP + ADP + H+	BB_0239/BB_0128/BB_0791/BB_0015	**0.00**	nf	–	nf	–
	DADE_K	Deoxyadenosine kinase	ATP + 2'-deoxyadenosine → ADP + dAMP + H+	BB_0239/BB_0791/BB_0015	**0.00**	nf	–	DADNK_1	1.00
	AMP_K	Adenylate kinase	ATP +AMP → 2 ADP	BB_0417	**0.00**	ADK1	1.00	ADK1/ADK2_1/ADKd^*c*^	1.00
	DAMP_K	Deoxyadenylate kinase	ATP + dAMP → ADP + dADP	BB_0417	**0.00**	DADK	1.00	DADK	1.00
	TMP_K	Thymidylate kinase	dTMP + ATP → ADP + dTDP	BB_0417/BB_0793	**0.00**	DTMPK	1.00	DTMPK	**0.00**
	CMP_K	Cytidylate kinase	CMP + ATP → ADP + CDP	BB_0417/BB_0128/BB_0819	**0.00**	CYTK1	1.00	CYTK1/CYTK1_1^*c*^	1.00
	DCMP_K	Deoxycytidylate kinase	dCMP + ATP → ADP + dCDP	BB_0417	**0.00**	CYTK2	1.00	CYTK2	0.00
	DGMP_K	Deoxyguanylate kinase	dGMP + ATP → ADP + dGDP	BB_0417	**0.00**	DGK1	1.00	DGK1/2	1.00

^*a*^ Reactions are predicted to be essential when an *in silico* deletion shows zero flux through the biomass reaction (growth ratio deletion:wild type = 0). Essential reactions highlighted bold. Most of the reactions shown are uniquely essential in *B. burgdorferi*, although some are also essential in *E. coli* or *S. aureus*. NF, reaction not found in organism: deletion growth ratio cannot be calculated (–) where reaction is not found. Complete list of essential reactions is found in supplemental file S5, “SRD.”

^*b*^ Any one of four mevalonate kinase reactions is required for growth.

^*c*^ No growth defect even if all homologs of adenylate/cytidylate kinase were deleted.

### Enzyme inhibitors prevent growth of *B. burgdorferi*

Four reactions with known small molecule inhibitors were assayed for growth inhibition in each of the three species. Cycloserine targets alanine racemase (60), an enzyme predicted to be essential for cell wall biosynthesis in all three species investigated. Bromopyruvate is an inhibitor of several enzymes in the glycolytic Embden-Meyerhof-Parnas (EMP) pathway (66), which is known to be essential in *S. aureus* (67) and is the only known ATP-generating pathway in *B. burgdorferi* (6). In *E. coli*, the Entner-Doudouroff glycolytic pathway and fatty acid beta oxidation are alternatives to EMP glycolysis, both generating acetyl-CoA to drive ATP production via the tricarboxylic acid cycle. Like *B. burgdorferi*, *S. aureus* lacks either of these alternatives (68).

Theophylline and pemetrexed target pyridoxal kinase (69) and serine hydroxymethltransferase (70), respectively. Both enzymes are predicted to be uniquely essential to *B. burgdorferi*: *E. coli* and *S. aureus* possess redundant pathways for the synthesis of the essential products pyridoxal phosphate and methylene-tetrahydrofolate.

**Fig 2:**
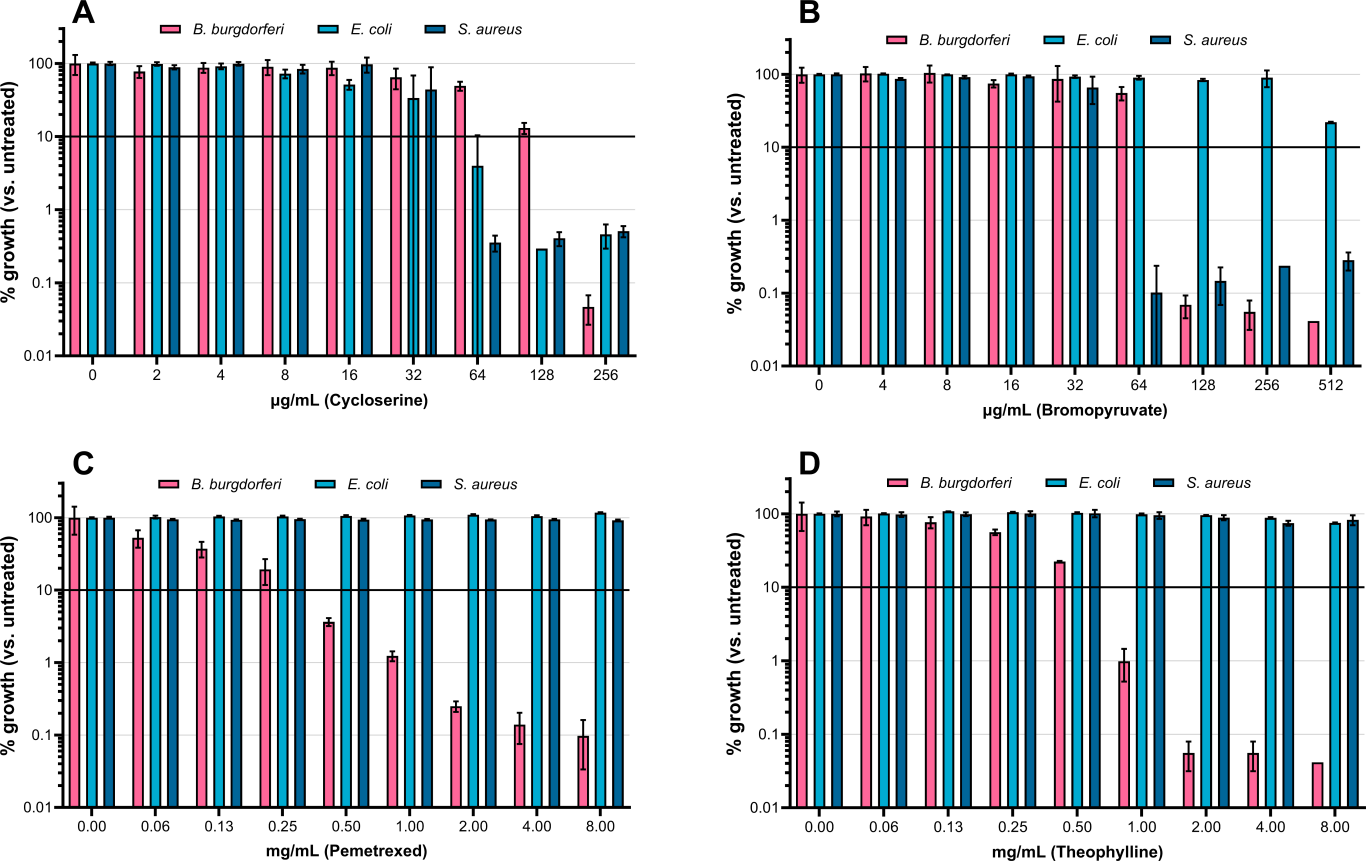
Inhibition of growth of three bacterial species by four small molecule inhibitors. D-cycloserine (**A**) prevents growth of all three species at similar concentrations. Bromopyruvate (**B**) inhibits the glycolysis-dependent species, *B. burgdorferi* and *S. aureus*. Both pemetrexed (**C**) and theophylline (**D**) selectively inhibit *B. burgdorferi. B. burgdorferi* growth quantified by direct cell counts (cells/mL); *E. coli* and *S. aureus* growth, by optical density (OD_600_): values for all three were normalized to growth of an untreated control grown in parallel. Black line represents 10% of the growth of untreated controls: the cutoff for determination of MIC.

Experimentally determined minimum inhibitory concentrations (MICs; Table 2) match the predictions of reaction essentiality (Fig. 2). Cycloserine effectively inhibits all three species with similar MIC. The experimental MIC of cycloserine is fourfold higher in *B. burgdorferi* in than the other species. This variation may be due to nonmetabolic differences: transport, efflux, or degradation of the drug may vary between the species. Bromopyruvate inhibits the growth of EMP glycolysis-dependent species *B. burgdorferi* (MIC = 128 µg/mL) and *S. aureus* (MIC = 64 µg/mL) at concentrations which do not inhibit *E. coli* (MIC >512 µg/mL). Both theophylline (MIC = 1 mg/mL) and pemetrexed (MIC = 0.5 mg/mL) have specific activity against *B. burgdorferi* without inhibiting growth of either *E. coli* or *S. aureus* in the concentration range tested (MIC >8 mg/mL).

**TABLE 2:** Predicted growth ratios (growth rate of knockout/growth rate of wild type, where 0 = no growth) and experimentally determined MIC for four reactions across three species^*a*^

Species (model)	Reaction (ID)	Predicted growth ratio (KO/WT)	Compound	MIC
*B. burgdorferi* (iBB151)	Alanine racemase (ALA_R)	0	D-cycloserine	**256** µg/mL
*E. coli* (iML1515)	Alanine racemase (ALAR)	0	D-cycloserine	**64** µg/mL
*S. aureus* (iYS854)	Alanine racemase (ALAR)	0	D-cycloserine	**64** µg/mL
*B. burgdorferi* (iBB151)	GAPDH (GAP_DH)	0	Bromopyruvate	**128** µg/mL
*E. coli* (iML1515)	GAPDH (GAPD)	0.87	Bromopyruvate	>512 µg/mL
*S. aureus* (iYS854)	GAPDH (GAPD_1)	0	Bromopyruvate	**64** µg/mL
*B. burgdorferi* (iBB151)	Pyridoxal kinase (PYDX_K)	0	Theophylline	**1** mg/mL
*E. coli* (iML1515)	Pyridoxal kinase (PYDXK)	1	Theophylline	>8 mg/mL
*S. aureus* (iYS854)	Pyridoxal kinase (PYDXK_1)	1	Theophylline	>8 mg/mL
*B. burgdorferi* (iBB151)	Serine hydroxymethyltransferase (SER_HMT)	0	Pemetrexed	**0.5** mg/mL
*E. coli* (iML1515)	Serine hydroxymethyltransferase (GHMT2r)	0.98	Pemetrexed	>8 mg/mL
*S. aureus* (iYS854)	Serine hydroxymethyltransferase (GHMT2r)	0.98	Pemetrexed	>8 mg/mL

^*a*^ Inhibitory concentrations were reached (highlighted bold) where *in silico* analysis predicted gene essentiality.

### Enzyme inhibitors reduce viability in established *B. burgdorferi* cultures

**Fig 3:**
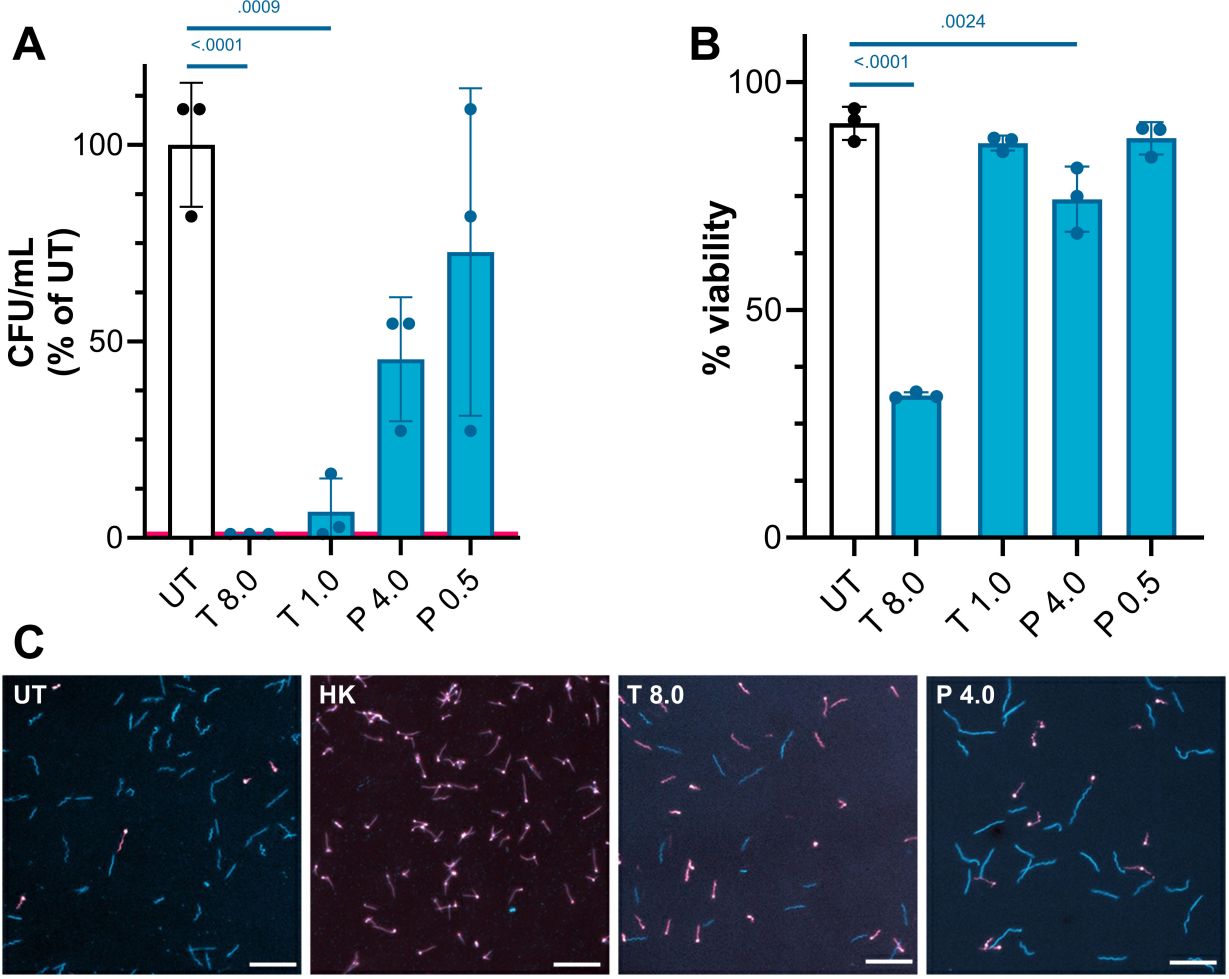
Killing of *Borrelia burgdorferi* by 20-h exposure to antibiotics. Cells were grown to stationary phase and incubated at 32°C for 20 hours with drugs (theophylline, T, and pemetrexed, P) at their MIC or eight times their MIC. Viability was normalized to that of untreated (UT) cells incubated in media only. After treatment, cells were diluted onto solid media overlay plates for enumeration of colony-forming units (**A**) or directly live/dead stained (**B**). Colonies were counted at 14 days: the lower limit of detection (pink line) was 2 × 10^5^ cells or a 99.8% reduction from 1 × 10^8^. At 8 mg/mL, all theophylline-treated cultures fell below the LOD. For live/dead staining, treated cells were immediately stained with SYTO 9 (Em 503 nm; shown blue) and propidium iodide (Em 615 nm; shown pink): all cells are stained by SYTO 9, while only dead cells with permeable membranes are stained by propidium iodide. Pink and blue cells were counted to yield % viable counts (**B**). *P*-values shown were calculated by one-way analysis of variance with Dunnett’s test: *P*-values not shown were >0.05. Example images in C show untreated (UT), heat-killed (HK), and theophylline (T 8.0)- and pemetrexed (P 4.0)-treated cells. Scale bars, 25 µm.

Estimation of viability in *B. burgdorferi* is complicated by the fact that the species forms neither turbid cultures nor traditional colonies. We determined the effect of candidate drugs on stationary-phase *B. burgdorferi* using three complementary methods (Fig. 3). Enumeration of “colonies” (visible growth in solid media overlays) after serial dilution (3A) revealed reduction of >99.8% in viable cell counts with theophylline at 8 mg/mL (8× the MIC) and reduction by 84% at the MIC, 1 mg/mL. Pemetrexed had less bactericidal efficacy: even at 8× the MIC viability was only reduced ~72%.

We also used a direct staining method to measure viability immediately after treatment. Propidium iodide is a membrane-impermeable dye and only stains cells in which membrane integrity has been lost, usually as a result of cell death. SYTO 9 is membrane permeable and stains viable cells in the absence of propidium iodide. This method produces lower estimates of killing than the CFU counting, but microscopy did reveal morphological changes after some treatments. Despite largely retaining membrane integrity, pemetrexed-treated cells were morphologically different (Fig. 3C), appearing longer and with a less regular helical shape. Greater viability in the direct staining assay may suggest that cell death occurs via a mechanism in which membrane integrity is not compromised or that treatment induces the formation of an intact and viable but slow-growing subpopulation (which appears dead in outgrowth assays but not by staining) in addition to killing some of the treated *Borrelia*. Both methods agree that treatment with theophylline at 8× the MIC has a significant bactericidal effect on stationary-phase *B. burgdorferi*. The lower concentrations of theophylline and pemetrexed at either concentration were less effective.

## DISCUSSION

The model generated here represents the most complete synthesis of predictive and experimental data available to date. While iBB151 may not completely describe the metabolism of *B. burgdorferi*, it was sufficient to generate experimentally validated predictions. The model produced here annotated 208 reactions to 151 genes. Although many of these genes are predicted by other pipelines such as KEGG (29) or the NCBI’s prokaryotic genome annotation pipeline (71), the integration of multiple such pipelines with experimental data makes iBB151 more comprehensive than any single existing database. Two pipelines cross-referenced the *B. burgdorferi* genome to existing annotation databases. The MetaCyc pipeline looked for homology to any enzyme in the MetaCyc database, whereas the HMM pipeline probed the KEGG database. The MetaCyc metabolism database is manually curated and, therefore, smaller than the automated KEGG, and accordingly, our MetaCyc pipeline predicted fewest reactions. The KEGG database includes automated annotations of over 9,000 organisms, providing a larger (but less curated) set of enzyme sequences for the HMM pipeline. Our third annotation, the KEGG pipeline, pulled directly from KEGG’s automated annotation (72) of the *B. burgdorferi* genome. As the HMM pipeline is based on KEGG’s database, it is unsurprising that these two pipelines generated models with significant overlap. Each pipeline annotated a different but overlapping set of reactions, although 71 of 208 reactions were predicted by all three pipelines. These 71 are likely well conserved between *Borrelia* and other better-described species and thus predicted with greater confidence.

Prediction of enzymatic functions by homology to other sequences is confounded by the evolutionary divergence of the spirochetes, which leaves a high proportion of the genome annotated only as hypothetical proteins. Complete characterization of these proteins will require further experimental approaches, but *B. burgdorferi* is a fastidious organism with a slow growth rate and complex genome; these factors combine to make traditional studies of metabolism challenging. By developing and analyzing the novel *in silico* metabolic network described here, 28 anti-borrelial drug targets were predicted. Existing small molecule drugs were repurposed to target two of these *B. burgdorferi* enzymes and shown to have selective activity in culture.

The prediction of essential reactions is highly dependent on the availability of extracellular metabolites, analogous to an *in silico* growth medium. *In vivo*, the essentiality of reactions predicted by modeling here could be overcome by the uptake of products from a rich extracellular environment. For instance, the phospholipid synthases are predicted to be essential but could become nonessential due to passive uptake where the environment is abundant in phospholipids (55). The production of the cell wall diamino acid ornithine by arginine deiminase (BB_0841) is predicted essential here, but the enzyme has previously been inactivated both singly (49) and as part of a transposon library (57). Thus, the prediction of arginine deiminase essentiality is likely to reflect only the requirement for ornithine, which can be sourced either through the arginine deiminase system or by direct uptake from the environment. A modification of the model to supply ornithine as an extracellular metabolite renders the reaction nonessential. Similarly, there are examples of genes dispensable in culture but essential during infection, such as the nicotinamidase *pncA* (BB_E22) (73, 74). Matching *in vitro* data, BB_E22 is predicted nonessential here, due to the presence of nicotinamide mononucleotide (NMN) in the synthetic growth medium. However, a double gene deletion of BB_E22 and nicotinamide mononucleotide transport (transport_59) is lethal—nicotinamide mononucleotide provides an alternative substrate for NAD synthesis via NMN adenyltransferase. This double deletion effectively recreates the BB_E22 deletion in an environment with no extracellular NMN—poor availability of NAD/NMN in mammalian hosts may be the reason for *in vivo* essentiality of BB_E22. These examples illustrate the importance of the *in silico* growth medium in prediction of essential reactions. Modification of the growth medium based on nutrient availability in the various niches of the infective cycle may yield site-specific predictions of gene essentiality.

Many of the 77 genes predicted as essential represent candidate targets for the development of novel anti-borrelial antibiotics. Repurposing four existing inhibitors of related enzymes, predictions were validated in culture and the inhibitors shown to have activity against *B. burgdorferi*, with three of those exhibiting a degree of specificity. D-cycloserine is used as part of combination therapies against *M. tuberculosis*, but the other compounds tested are unsuitable for clinical use. Although tested extensively against cultured cancer cells, bromopyruvate has not been studied in clinical trials: its simple structure and broad range of cellular targets (75) raise concerns of off-target toxicity. Theophylline is rarely used for its original indication against asthma due to a narrow therapeutic index and reported toxicity (76). Like many anticancer drugs, pemetrexed also has a narrow therapeutic window and has significant associated toxicity (77). While the inhibitory concentrations determined here for theophylline and pemetrexed (1.0 mg/mL and 0.5 mg/mL) are high, efficacy is likely to be improved by modification of these repurposed drugs (both developed to target mammalian enzyme homologs) to bind their *B. burgdorferi* enzyme targets more efficiently and specifically. These modifications, and others to improve bioavailability and activity, will be the focus of future work. The ability of theophylline to kill even stationary-phase *Borrelia* suggests the possibility that the targeting of core metabolism may be effective even in established or persistent infections, where traditional antibiotics have limited efficacy.

The study of core metabolism is especially important in host-dependent pathogens such as *B. burgdorferi*, where the complex metabolic interactions between pathogen and host underpin persistence and pathogenesis. Furthermore, the *in silico* approach to drug target identification described here could be of particular value when applied to other fastidious organisms for which experimental approaches are difficult. The metabolic model generated may also facilitate further study of the core metabolism of *B. burgdorferi* and the complex interactions between the bacterium and its various animal hosts. Four essential reactions are validated here *in vitro*, and the two candidates for the development of specific anti-borrelial drugs will be further investigated to design more effective and tolerable inhibitors. Development of these targets into narrow-spectrum antimicrobials could reduce the incidence of Lyme disease by reservoir-targeted elimination of *B. burgdorferi* or as prophylaxis in high-risk groups.

## SUPPLEMENTAL MATERIAL

File S1 msystems.00835-23-s0001.docx Legends describing the other supplemental files.

File S2 msystems.00835-23-s0002.pdf Metabolic model overview showing essential reactions.

File S3 msystems.00835-23-s0003.pdf Metabolic model overview highlighting major pathways.

File S4 msystems.00835-23-s0004.xlsx Metabolic model in Excel format.

File S5 msystems.00835-23-s0005.xlsx Predictions of essential reactions and genes.

ASM does not own the copyrights to Supplemental Material that may be linked to, or accessed through, an article. The authors have granted ASM a non-exclusive, world-wide license to publish the Supplemental Material files. Please contact the corresponding author directly for reuse.
