# Supplementary material for: Metabolic modeling predicts unique drug targets in Borrelia burgdorferi
Source: mSystems. 2023 Oct 19;8(6):e00835-23. doi: 10.1128/msystems.00835-23 (PMC10734484; doi:10.1128/msystems.00835-23)
Supplement: File S1 — Legends describing the other supplemental files. [file msystems.00835-23-s0001.docx]

**Supplemental file 2**

Visualization of iBB151 generated in Cytoscape v3.9.1 showing essential reactions. Extracellular metabolites are hollow circles, intracellular metabolites blue circles, and biomass precursors pink. Enzymatic reactions represented as lines, with essential reactions highlighted pink. Gene associations for each reaction are shown where known.

**Supplemental file 3**

Visualization of iBB151 generated in Cytoscape v3.9.1 with pathways highlighted. Extracellular metabolites are hollow circles, intracellular metabolites filled circles, and biomass precursors larger circles. Enzymatic reactions represented as lines, with gene associations for each reaction shown where known.

**Supplemental file 4**

iBB151 model file in excel format. RXNS, METS, COMPS, GENES, and MODEL tabs are part of standard metabolic model formatting and required for parsing into analysis packages such as RAVEN and COBRA. Essential Rxns, Single Gene Deletion, and Double Gene Deletion are informational only.

**RXNS** tab:

**ID**: a short reaction identifier, unique to this model.

**NAME**: longer enzyme name (or names)

**EQUATION**: reactants and products for the reaction. Square brackets refer to the compartment each metabolite is in. => or <= are irreversible reactions, <=> are irreversible.

**EC-NUMBER**: enzyme commission reference number for the enzyme.

**GENE ASSOCIATION**: gene or genes responsible for the reaction. Where two genes are listed, they are redundant enzymes.

**LOWER BOUND**: the minimum flux through the reaction (left to right). Set to 0 for irreversible reactions, and -1000 for reversible reactions. To inactivate a reaction, lower and upper bounds are both set to 0.

**UPPER BOUND**: the maximum flux through the reaction (left to right). To inactivate a reaction, lower and upper bounds are both set to 0.

**OBJ**: the objective function of the model is marked with 1. Set to EX_BM as default, but can be changed. There can only be one objective function.

**COMP**: the compartment in which the reaction occurs. Left blank here as we only included two compartments: all reactions are considered cytoplasmic.

**From KEGG/Metacyc/HMM**: the annotation pipeline in which the reaction/enzyme/gene was predicted: many were predicted in more than one pipeline.

**Manually Curated**: X if the reaction was not predicted but included based on data from the literature.

**NOTE**: any extra information

**REFERENCE**: DOIs for reactions based on literature.

**MIRIAM**: references to the same reaction in other databases, where available.

**SUBSYSTEM/REPLACEMENT ID/CONFIDENCE SCORE**: used in some metabolic models but not used here.

**METS** tab:

**ID**: metabolite name as it appears in reactions.

**NAME**: full name of metabolite

**UNCONSTRAINED**: used to define boundary metabolites in some models, but not used here

**MIRIAM**: references to the same metabolite in other databases, where available

**COMPOSITION**: chemical formula of the metabolite, used for mass balancing

**InChl**: not used here as the information is contained in the MIRIAM column

**COMPARTMENT**: the compartment in which the metabolite is found.

**REPLACEMENT ID**: a shorter name

**CHARGE**: not included here because we didn’t attempt charge balancing

**COMPS** tab:

**ABBREVIATION**: how the compartment is referenced elsewhere

**NAME**: name of the compartment

**INSIDE**: not used here as we only have two compartments

**MIRIAM**: not used here

**GENES** tab:

**NAME**: gene name: either the BB_ number, or a placeholder name where the gene is unknown

**MIRIAM**: references to the same gene in other databases, where available.

**SHORT NAME**: a shorter gene name

**COMPARTMENT**: not used here

**MODEL** tab:

**ID**: the model name

**DESCRIPTION**: organism name

**TAXONOMY**: NCBI taxonomy and genbank assembly accession numbers

**DEFAULT LOWER/UPPER**: lower and upper bounds for any reaction where these are not specified

**Supplemental file 5**

Containing predictions of reaction and gene essentiality.

**Essential Rxns** tab:

**ID/EQUATION/GENE ASSOCIATION**: copied from the RXNS tab

**hasFlux?**: whether the reaction is able to carry flux under the default conditions, determined using the RAVEN command hasFlux. This should be TRUE for every reaction.

**sol.x**: the flux through the reaction when solved under the default conditions

**gRate**: growth rate (flux through the objective function, EX_BM) when the reaction is inactivated as determined using the COBRA command singleRxnDeletion

**gRatio**: growth ratio (growth rate of mutant/growth rate of wild type) as determined using the COBRA command singleRxnDeletion

**Single Gene Deletion** tab:

**NAME**: gene name

**gRate**: growth rate (flux through the objective function, EX_BM) when the gene is deleted as determined using the COBRA command singleGeneDeletion

**gRatio**: growth ratio (growth rate of mutant/growth rate of wild type) as determined using the COBRA command singleGeneDeletion

**isEssential**: 1 if the gene is essential, 0 if it isn’t

**REDUNDANCIES**: other genes predicted carry out the same reaction(s)

**hasTn**: whether a transposon insertion was found in a previous mutagenesis screen (Lin et al 2012: <https://doi.org/10.1371/journal.pone.0047532>)

**Correct Call**: some simple arithmetic transformations to cross-reference the binary data in isEssential and hasTn and determine accuracy of the prediction of essentiality

**Double Gene Deletion** tab:

Matrix showing the growth ratios (growth rate of double mutant/growth rate of wild type, as determined using the COBRA command doubleGeneDeletion) of selected double deletions. For simplicity, any gene that was single-deletion lethal or not lethal in any combination was removed.
