# Supplementary material for: Metabolic modeling predicts unique drug targets in Borrelia burgdorferi
Source: mSystems. 2023 Oct 19;8(6):e00835-23. doi: 10.1128/msystems.00835-23 (PMC10734484; doi:10.1128/msystems.00835-23)
Supplement: File S2 — Metabolic model overview showing essential reactions. [file msystems.00835-23-s0002.pdf]

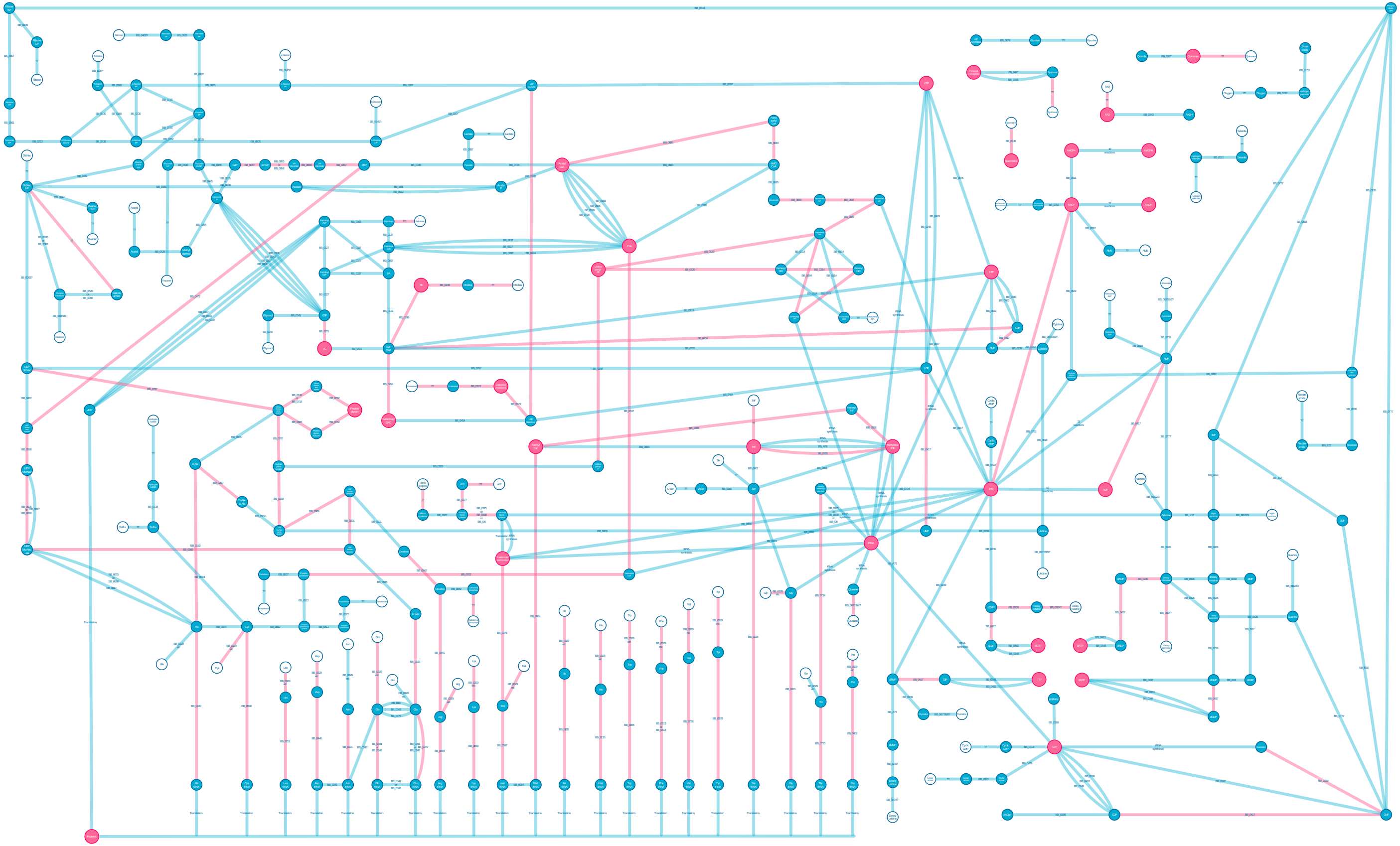

Supplemental Figure 1.
Representation of iBB151, the metabolic model of Borrelia burgdorferi. Blue circles: intracellular metabolites, white circles: extracellular metabolites, pink circles: biomass components. Lines represent enzymatic reactions, with gene associations for each reaction also shown. Pink lines are reactions predicted to be essential.
