# Supplementary material for: Metabolic modeling predicts unique drug targets in Borrelia burgdorferi
Source: mSystems. 2023 Oct 19;8(6):e00835-23. doi: 10.1128/msystems.00835-23 (PMC10734484; doi:10.1128/msystems.00835-23)
Supplement: File S3 — Metabolic model overview highlighting major pathways. [file msystems.00835-23-s0003.pdf]

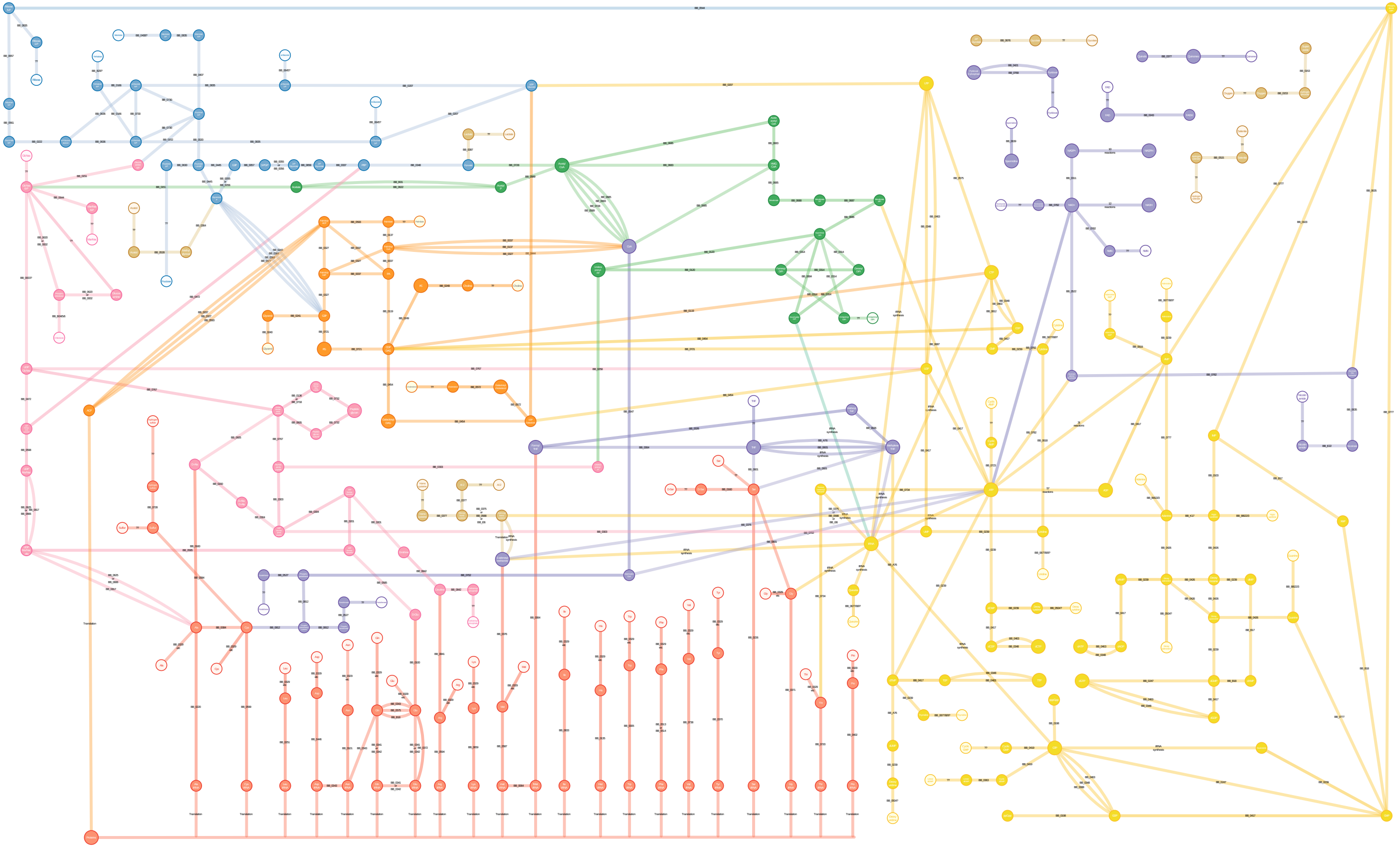

Supplemental figure 2.
Representation of iBB151, the metabolic model of Borrelia burgdorferi. Filled circles: intracellular metabolites, hollow circles: extracellular metabolites, large circles: biomass components. Lines represent enzymatic reactions, with gene associations for each reaction also shown. Reactions colored by pathway. Blue: carbon metabolism and glycolysis. Pink: peptidoglycan synthesis: Orange: lipids. Red: amino acids and translation. Purple: cofactor synthesis. Green: mevalonate pathway. Yellow: nucleotides. Brown: detoxification systems.
